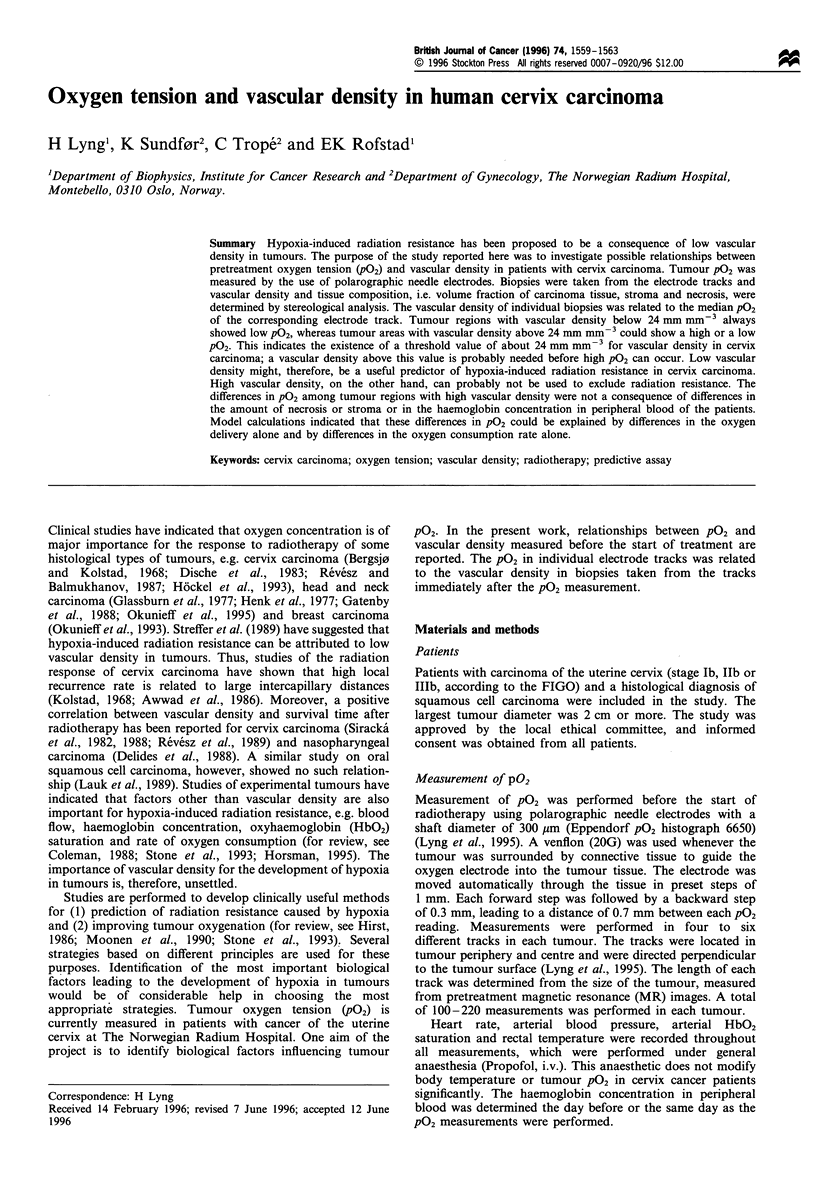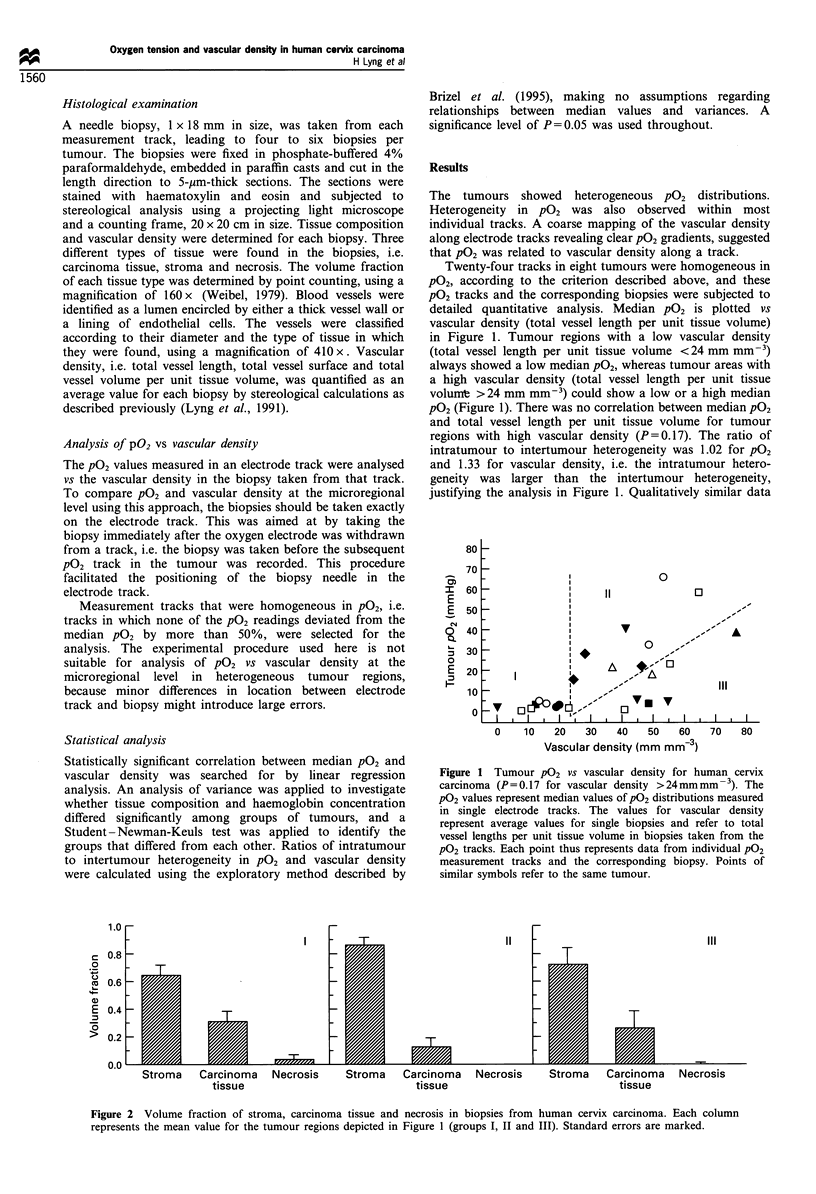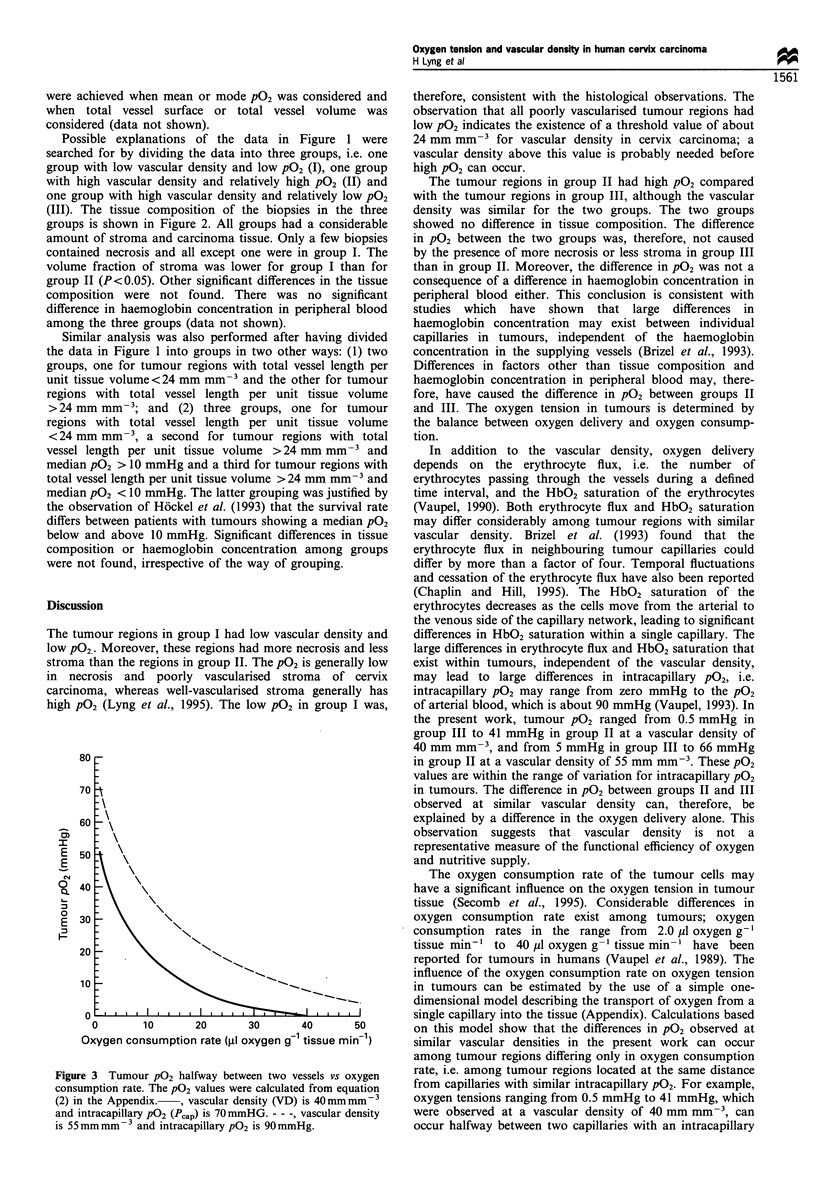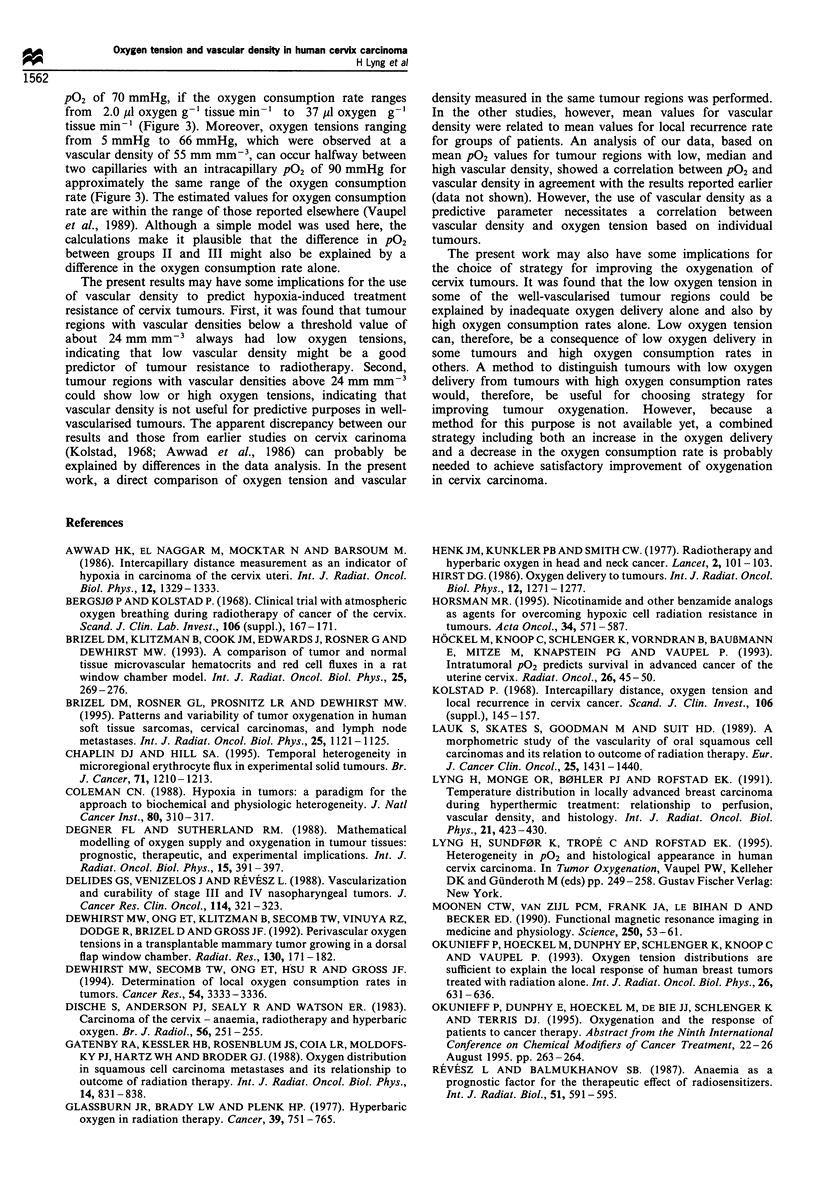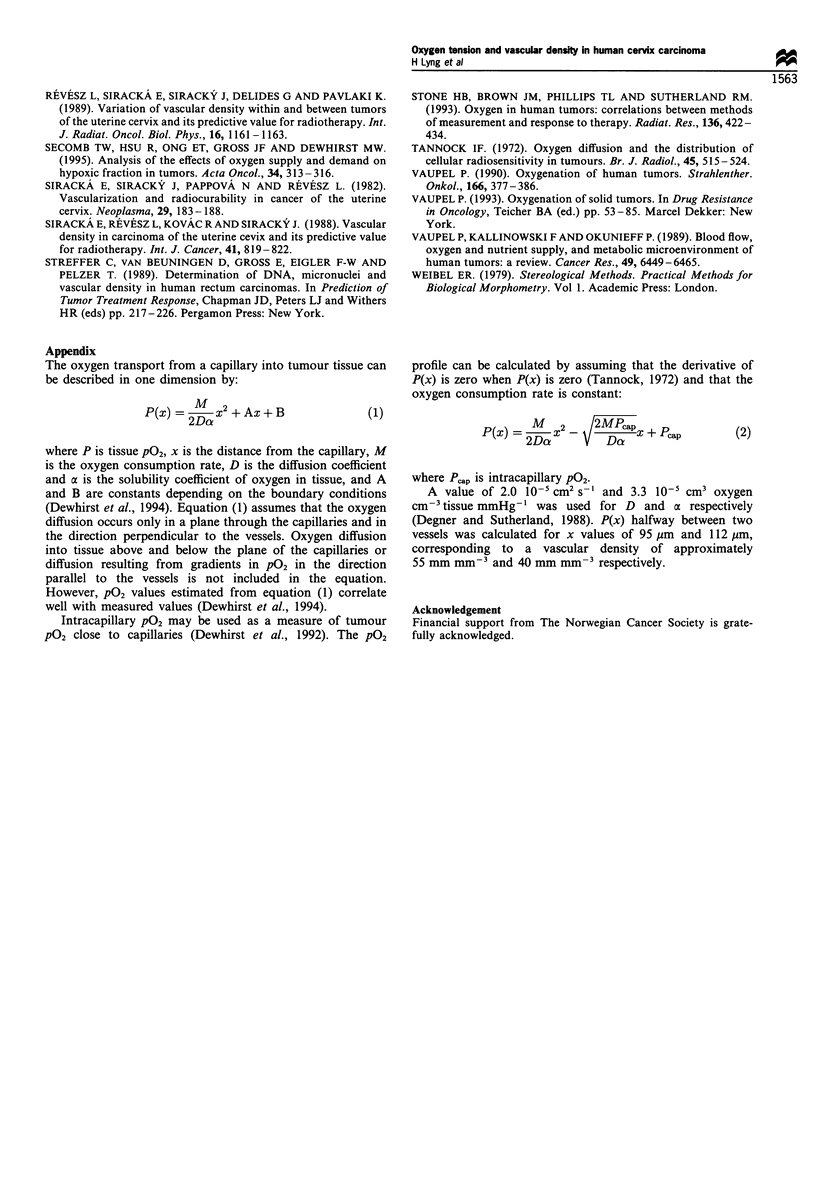# Oxygen tension and vascular density in human cervix carcinoma.

**DOI:** 10.1038/bjc.1996.589

**Published:** 1996-11

**Authors:** H. Lyng, K. Sundfør, C. Tropé, E. K. Rofstad

**Affiliations:** Department of Biophysics, Institute for Cancer Research, Oslo, Norway.

## Abstract

Hypoxia-induced radiation resistance has been proposed to be a consequence of low vascular density in tumours. The purpose of the study reported here was to investigate possible relationships between pretreatment oxygen tension (pO2) and vascular density in patients with cervix carcinoma. Tumour pO2 was measured by the use of polarographic needle electrodes. Biopsies were taken from the electrode tracks and vascular density and tissue composition, i.e. volume fraction of carcinoma tissue, stroma and necrosis, were determined by stereological analysis. The vascular density of individual biopsies was related to the median pO2 of the corresponding electrode track. Tumour regions with vascular density below 24 mm mm(-3) always showed low pO2, whereas tumour areas with vascular density above 24 mm mm(-3) could show a high or a low pO2. This indicates the existence of a threshold value of about 24 mm mm(-3) for vascular density in cervix carcinoma; a vascular density above this value is probably needed before high pO2 can occur. Low vascular density might, therefore, be a useful predictor of hypoxia-induced radiation resistance in cervix carcinoma. High vascular density, on the other hand, can probably not be used to exclude radiation resistance. The differences in pO2 among tumour regions with high vascular density were not a consequence of differences in the amount of necrosis or stroma or in the haemoglobin concentration in peripheral blood of the patients. Model calculations indicated that these differences in pO2 could be explained by differences in the oxygen delivery alone and by differences in the oxygen consumption rate alone.


British Journal of Cancer (1996) 74, 1559-1563
© 1996 Stockton Press All rights reserved 0007-0920/96 $12.00

### Oxygen tension and vascular density in human cervix carcinoma

H Lyng[1], K Sundfør[2], C Tropé[2] and EK Rofstad[1]

[1]*Department of Biophysics, Institute for Cancer Research and* [2]*Department of Gynecology, The Norwegian Radium Hospital, Montebello, 0310 Oslo, Norway.*

**Summary** Hypoxia-induced radiation resistance has been proposed to be a consequence of low vascular density in tumours. The purpose of the study reported here was to investigate possible relationships between pretreatment oxygen tension ($pO_2$) and vascular density in patients with cervix carcinoma. Tumour $pO_2$ was measured by the use of polarographic needle electrodes. Biopsies were taken from the electrode tracks and vascular density and tissue composition, i.e. volume fraction of carcinoma tissue, stroma and necrosis, were determined by stereological analysis. The vascular density of individual biopsies was related to the median $pO_2$ of the corresponding electrode track. Tumour regions with vascular density below 24 mm $mm^{-3}$ always showed low $pO_2$, whereas tumour areas with vascular density above 24 mm $mm^{-3}$ could show a high or a low $pO_2$. This indicates the existence of a threshold value of about 24 mm $mm^{-3}$ for vascular density in cervix carcinoma; a vascular density above this value is probably needed before high $pO_2$ can occur. Low vascular density might, therefore, be a useful predictor of hypoxia-induced radiation resistance in cervix carcinoma. High vascular density, on the other hand, can probably not be used to exclude radiation resistance. The differences in $pO_2$ among tumour regions with high vascular density were not a consequence of differences in the amount of necrosis or stroma or in the haemoglobin concentration in peripheral blood of the patients. Model calculations indicated that these differences in $pO_2$ could be explained by differences in the oxygen delivery alone and by differences in the oxygen consumption rate alone.

**Keywords:** cervix carcinoma; oxygen tension; vascular density; radiotherapy; predictive assay

Clinical studies have indicated that oxygen concentration is of major importance for the response to radiotherapy of some histological types of tumours, e.g. cervix carcinoma (Bergsjø and Kolstad, 1968; Dische *et al.*, 1983; Révész and Balmukhanov, 1987; Höckel *et al.*, 1993), head and neck carcinoma (Glassburn *et al.*, 1977; Henk *et al.*, 1977; Gatenby *et al.*, 1988; Okunieff *et al.*, 1995) and breast carcinoma (Okunieff *et al.*, 1993). Streffer *et al.* (1989) have suggested that hypoxia-induced radiation resistance can be attributed to low vascular density in tumours. Thus, studies of the radiation response of cervix carcinoma have shown that high local recurrence rate is related to large intercapillary distances (Kolstad, 1968; Awwad *et al.*, 1986). Moreover, a positive correlation between vascular density and survival time after radiotherapy has been reported for cervix carcinoma (Siracká *et al.*, 1982, 1988; Révész *et al.*, 1989) and nasopharyngeal carcinoma (Delides *et al.*, 1988). A similar study on oral squamous cell carcinoma, however, showed no such relationship (Lauk *et al.*, 1989). Studies of experimental tumours have indicated that factors other than vascular density are also important for hypoxia-induced radiation resistance, e.g. blood flow, haemoglobin concentration, oxyhaemoglobin ($HbO_2$) saturation and rate of oxygen consumption (for review, see Coleman, 1988; Stone *et al.*, 1993; Horsman, 1995). The importance of vascular density for the development of hypoxia in tumours is, therefore, unsettled.

Studies are performed to develop clinically useful methods for (1) prediction of radiation resistance caused by hypoxia and (2) improving tumour oxygenation (for review, see Hirst, 1986; Moonen *et al.*, 1990; Stone *et al.*, 1993). Several strategies based on different principles are used for these purposes. Identification of the most important biological factors leading to the development of hypoxia in tumours would be of considerable help in choosing the most appropriate strategies. Tumour oxygen tension ($pO_2$) is currently measured in patients with cancer of the uterine cervix at The Norwegian Radium Hospital. One aim of the project is to identify biological factors influencing tumour $pO_2$. In the present work, relationships between $pO_2$ and vascular density measured before the start of treatment are reported. The $pO_2$ in individual electrode tracks was related to the vascular density in biopsies taken from the tracks immediately after the $pO_2$ measurement.

Correspondence: H Lyng
Received 14 February 1996; revised 7 June 1996; accepted 12 June 1996

### Materials and methods

### *Patients*

Patients with carcinoma of the uterine cervix (stage Ib, IIb or IIIb, according to the FIGO) and a histological diagnosis of squamous cell carcinoma were included in the study. The largest tumour diameter was 2 cm or more. The study was approved by the local ethical committee, and informed consent was obtained from all patients.

### *Measurement of* $pO_2$

Measurement of $pO_2$ was performed before the start of radiotherapy using polarographic needle electrodes with a shaft diameter of 300 $\mu$m (Eppendorf $pO_2$ histograph 6650) (Lyng *et al.*, 1995). A venflon (20G) was used whenever the tumour was surrounded by connective tissue to guide the oxygen electrode into the tumour tissue. The electrode was moved automatically through the tissue in preset steps of 1 mm. Each forward step was followed by a backward step of 0.3 mm, leading to a distance of 0.7 mm between each $pO_2$ reading. Measurements were performed in four to six different tracks in each tumour. The tracks were located in tumour periphery and centre and were directed perpendicular to the tumour surface (Lyng *et al.*, 1995). The length of each track was determined from the size of the tumour, measured from pretreatment magnetic resonance (MR) images. A total of 100-220 measurements was performed in each tumour.

Heart rate, arterial blood pressure, arterial $HbO_2$ saturation and rectal temperature were recorded throughout all measurements, which were performed under general anaesthesia (Propofol, i.v.). This anaesthetic does not modify body temperature or tumour $pO_2$ in cervix cancer patients significantly. The haemoglobin concentration in peripheral blood was determined the day before or the same day as the $pO_2$ measurements were performed.

### *Histological examination*

A needle biopsy, 1 × 18 mm in size, was taken from each measurement track, leading to four to six biopsies per tumour. The biopsies were fixed in phosphate-buffered 4% paraformaldehyde, embedded in paraffin casts and cut in the length direction to 5-$\mu$m-thick sections. The sections were stained with haematoxylin and eosin and subjected to stereological analysis using a projecting light microscope and a counting frame, 20 × 20 cm in size. Tissue composition and vascular density were determined for each biopsy. Three different types of tissue were found in the biopsies, i.e. carcinoma tissue, stroma and necrosis. The volume fraction of each tissue type was determined by point counting, using a magnification of 160 × (Weibel, 1979). Blood vessels were identified as a lumen encircled by either a thick vessel wall or a lining of endothelial cells. The vessels were classified according to their diameter and the type of tissue in which they were found, using a magnification of 410 ×. Vascular density, i.e. total vessel length, total vessel surface and total vessel volume per unit tissue volume, was quantified as an average value for each biopsy by stereological calculations as described previously (Lyng *et al*., 1991).

### *Analysis of* p$O_2$ vs *vascular density*

The $pO_2$ values measured in an electrode track were analysed *vs* the vascular density in the biopsy taken from that track. To compare $pO_2$ and vascular density at the microregional level using this approach, the biopsies should be taken exactly on the electrode track. This was aimed at by taking the biopsy immediately after the oxygen electrode was withdrawn from a track, i.e. the biopsy was taken before the subsequent $pO_2$ track in the tumour was recorded. This procedure facilitated the positioning of the biopsy needle in the electrode track.

Measurement tracks that were homogeneous in $pO_2$, i.e. tracks in which none of the $pO_2$ readings deviated from the median $pO_2$ by more than 50%, were selected for the analysis. The experimental procedure used here is not suitable for analysis of $pO_2$ *vs* vascular density at the microregional level in heterogeneous tumour regions, because minor differences in location between electrode track and biopsy might introduce large errors.

### *Statistical analysis*

Statistically significant correlation between median $pO_2$ and vascular density was searched for by linear regression analysis. An analysis of variance was applied to investigate whether tissue composition and haemoglobin concentration differed significantly among groups of tumours, and a Student–Newman-Keuls test was applied to identify the groups that differed from each other. Ratios of intratumour to intertumour heterogeneity in $pO_2$ and vascular density were calculated using the exploratory method described by Brizel *et al.* (1995), making no assumptions regarding relationships between median values and variances. A significance level of $P = 0.05$ was used throughout.

### Results

The tumours showed heterogeneous $pO_2$ distributions. Heterogeneity in $pO_2$ was also observed within most individual tracks. A coarse mapping of the vascular density along electrode tracks revealing clear $pO_2$ gradients, suggested that $pO_2$ was related to vascular density along a track.

Twenty-four tracks in eight tumours were homogeneous in $pO_2$, according to the criterion described above, and these $pO_2$ tracks and the corresponding biopsies were subjected to detailed quantitative analysis. Median $pO_2$ is plotted *vs* vascular density (total vessel length per unit tissue volume) in Figure 1. Tumour regions with a low vascular density (total vessel length per unit tissue volume $< 24$ mm mm$^{-3}$) always showed a low median $pO_2$, whereas tumour areas with a high vascular density (total vessel length per unit tissue volumte $> 24$ mm mm$^{-3}$) could show a low or a high median $pO_2$ (Figure 1). There was no correlation between median $pO_2$ and total vessel length per unit tissue volume for tumour regions with high vascular density ($P = 0.17$). The ratio of intratumour to intertumour heterogeneity was 1.02 for $pO_2$ and 1.33 for vascular density, i.e. the intratumour heterogeneity was larger than the intertumour heterogeneity, justifying the analysis in Figure 1. Qualitatively similar data

**Figure 1** Tumour $pO_2$ *vs* vascular density for human cervix carcinoma ($P = 0.17$ for vascular density $> 24$ mm mm$^{-3}$). The $pO_2$ values represent median values of $pO_2$ distributions measured in single electrode tracks. The values for vascular density represent average values for single biopsies and refer to total vessel lengths per unit tissue volume in biopsies taken from the $pO_2$ tracks. Each point thus represents data from individual $pO_2$ measurement tracks and the corresponding biopsy. Points of similar symbols refer to the same tumour.

**Figure 2** Volume fraction of stroma, carcinoma tissue and necrosis in biopsies from human cervix carcinoma. Each column represents the mean value for the tumour regions depicted in Figure 1 (groups I, II and III). Standard errors are marked.

were achieved when mean or mode $pO_2$ was considered and when total vessel surface or total vessel volume was considered (data not shown).

Possible explanations of the data in Figure 1 were searched for by dividing the data into three groups, i.e. one group with low vascular density and low $pO_2$ (I), one group with high vascular density and relatively high $pO_2$ (II) and one group with high vascular density and relatively low $pO_2$ (III). The tissue composition of the biopsies in the three groups is shown in Figure 2. All groups had a considerable amount of stroma and carcinoma tissue. Only a few biopsies contained necrosis and all except one were in group I. The volume fraction of stroma was lower for group I than for group II ($P<0.05$). Other significant differences in the tissue composition were not found. There was no significant difference in haemoglobin concentration in peripheral blood among the three groups (data not shown).

Similar analysis was also performed after having divided the data in Figure 1 into groups in two other ways: (1) two groups, one for tumour regions with total vessel length per unit tissue volume $<24$ mm mm$^{-3}$ and the other for tumour regions with total vessel length per unit tissue volume $>24$ mm mm$^{-3}$; and (2) three groups, one for tumour regions with total vessel length per unit tissue volume $<24$ mm mm$^{-3}$, a second for tumour regions with total vessel length per unit tissue volume $>24$ mm mm$^{-3}$ and median $pO_2$ $>10$ mmHg and a third for tumour regions with total vessel length per unit tissue volume $>24$ mm mm$^{-3}$ and median $pO_2$ $<10$ mmHg. The latter grouping was justified by the observation of Höckel *et al.* (1993) that the survival rate differs between patients with tumours showing a median $pO_2$ below and above 10 mmHg. Significant differences in tissue composition or haemoglobin concentration among groups were not found, irrespective of the way of grouping.

### Discussion

The tumour regions in group I had low vascular density and low $pO_2$.. Moreover, these regions had more necrosis and less stroma than the regions in group II. The $pO_2$ is generally low in necrosis and poorly vascularised stroma of cervix carcinoma, whereas well-vascularised stroma generally has high $pO_2$ (Lyng *et al.*, 1995). The low $pO_2$ in group I was, therefore, consistent with the histological observations. The observation that all poorly vascularised tumour regions had low $pO_2$ indicates the existence of a threshold value of about 24 mm mm$^{-3}$ for vascular density in cervix carcinoma; a vascular density above this value is probably needed before high $pO_2$ can occur.

The tumour regions in group II had high $pO_2$ compared with the tumour regions in group III, although the vascular density was similar for the two groups. The two groups showed no difference in tissue composition. The difference in $pO_2$ between the two groups was, therefore, not caused by the presence of more necrosis or less stroma in group III than in group II. Moreover, the difference in $pO_2$ was not a consequence of a difference in haemoglobin concentration in peripheral blood either. This conclusion is consistent with studies which have shown that large differences in haemoglobin concentration may exist between individual capillaries in tumours, independent of the haemoglobin concentration in the supplying vessels (Brizel *et al.*, 1993). Differences in factors other than tissue composition and haemoglobin concentration in peripheral blood may, therefore, have caused the difference in $pO_2$ between groups II and III. The oxygen tension in tumours is determined by the balance between oxygen delivery and oxygen consumption.

In addition to the vascular density, oxygen delivery depends on the erythrocyte flux, i.e. the number of erythrocytes passing through the vessels during a defined time interval, and the $HbO_2$ saturation of the erythrocytes (Vaupel, 1990). Both erythrocyte flux and $HbO_2$ saturation may differ considerably among tumour regions with similar vascular density. Brizel *et al.* (1993) found that the erythrocyte flux in neighbouring tumour capillaries could differ by more than a factor of four. Temporal fluctuations and cessation of the erythrocyte flux have also been reported (Chaplin and Hill, 1995). The $HbO_2$ saturation of the erythrocytes decreases as the cells move from the arterial to the venous side of the capillary network, leading to significant differences in $HbO_2$ saturation within a single capillary. The large differences in erythrocyte flux and $HbO_2$ saturation that exist within tumours, independent of the vascular density, may lead to large differences in intracapillary $pO_2$, i.e. intracapillary $pO_2$ may range from zero mmHg to the $pO_2$ of arterial blood, which is about 90 mmHg (Vaupel, 1993). In the present work, tumour $pO_2$ ranged from 0.5 mmHg in group III to 41 mmHg in group II at a vascular density of 40 mm mm$^{-3}$, and from 5 mmHg in group III to 66 mmHg in group II at a vascular density of 55 mm mm$^{-3}$. These $pO_2$ values are within the range of variation for intracapillary $pO_2$ in tumours. The difference in $pO_2$ between groups II and III observed at similar vascular density can, therefore, be explained by a difference in the oxygen delivery alone. This observation suggests that vascular density is not a representative measure of the functional efficiency of oxygen and nutritive supply.

The oxygen consumption rate of the tumour cells may have a significant influence on the oxygen tension in tumour tissue (Secomb *et al.*, 1995). Considerable differences in oxygen consumption rate exist among tumours; oxygen consumption rates in the range from 2.0 $\mu$l oxygen g$^{-1}$ tissue min$^{-1}$ to 40 $\mu$l oxygen g$^{-1}$ tissue min$^{-1}$ have been reported for tumours in humans (Vaupel *et al.*, 1989). The influence of the oxygen consumption rate on oxygen tension in tumours can be estimated by the use of a simple one-dimensional model describing the transport of oxygen from a single capillary into the tissue (Appendix). Calculations based on this model show that the differences in $pO_2$ observed at similar vascular densities in the present work can occur among tumour regions differing only in oxygen consumption rate, i.e. among tumour regions located at the same distance from capillaries with similar intracapillary $pO_2$. For example, oxygen tensions ranging from 0.5 mmHg to 41 mmHg, which were observed at a vascular density of 40 mm mm$^{-3}$, can occur halfway between two capillaries with an intracapillary

**Figure 3** Tumour $pO_2$ halfway between two vessels *vs* oxygen consumption rate. The $pO_2$ values were calculated from equation (2) in the Appendix.——, vascular density (VD) is 40 mm mm$^{-3}$ and intracapillary $pO_2$ ($P_{cap}$) is 70 mmHG. - - -, vascular density is 55 mm mm$^{-3}$ and intracapillary $pO_2$ is 90 mmHg.

$pO_2$ of 70 mmHg, if the oxygen consumption rate ranges from 2.0 $\mu$l oxygen $g^{-1}$ tissue $min^{-1}$ to 37 $\mu$l oxygen $g^{-1}$ tissue $min^{-1}$ (Figure 3). Moreover, oxygen tensions ranging from 5 mmHg to 66 mmHg, which were observed at a vascular density of 55 mm $mm^{-3}$, can occur halfway between two capillaries with an intracapillary $pO_2$ of 90 mmHg for approximately the same range of the oxygen consumption rate (Figure 3). The estimated values for oxygen consumption rate are within the range of those reported elsewhere (Vaupel *et al.*, 1989). Although a simple model was used here, the calculations make it plausible that the difference in $pO_2$ between groups II and III might also be explained by a difference in the oxygen consumption rate alone.

The present results may have some implications for the use of vascular density to predict hypoxia-induced treatment resistance of cervix tumours. First, it was found that tumour regions with vascular densities below a threshold value of about 24 mm $mm^{-3}$ always had low oxygen tensions, indicating that low vascular density might be a good predictor of tumour resistance to radiotherapy. Second, tumour regions with vascular densities above 24 mm $mm^{-3}$ could show low or high oxygen tensions, indicating that vascular density is not useful for predictive purposes in well-vascularised tumours. The apparent discrepancy between our results and those from earlier studies on cervix carinoma (Kolstad, 1968; Awwad *et al.*, 1986) can probably be explained by differences in the data analysis. In the present work, a direct comparison of oxygen tension and vascular density measured in the same tumour regions was performed. In the other studies, however, mean values for vascular density were related to mean values for local recurrence rate for groups of patients. An analysis of our data, based on mean $pO_2$ values for tumour regions with low, median and high vascular density, showed a correlation between $pO_2$ and vascular density in agreement with the results reported earlier (data not shown). However, the use of vascular density as a predictive parameter necessitates a correlation between vascular density and oxygen tension based on individual tumours.

The present work may also have some implications for the choice of strategy for improving the oxygenation of cervix tumours. It was found that the low oxygen tension in some of the well-vascularised tumour regions could be explained by inadequate oxygen delivery alone and also by high oxygen consumption rates alone. Low oxygen tension can, therefore, be a consequence of low oxygen delivery in some tumours and high oxygen consumption rates in others. A method to distinguish tumours with low oxygen delivery from tumours with high oxygen consumption rates would, therefore, be useful for choosing strategy for improving tumour oxygenation. However, because a method for this purpose is not available yet, a combined strategy including both an increase in the oxygen delivery and a decrease in the oxygen consumption rate is probably needed to achieve satisfactory improvement of oxygenation in cervix carcinoma.

### References

AWWAD HK, EL NAGGAR M, MOCKTAR N AND BARSOUM M. (1986). Intercapillary distance measurement as an indicator of hypoxia in carcinoma of the cervix uteri. *Int. J. Radiat. Oncol. Biol. Phys.*, **12**, 1329–1333.

BERGSJØ P AND KOLSTAD P. (1968). Clinical trial with atmospheric oxygen breathing during radiotherapy of cancer of the cervix. *Scand. J. Clin. Lab. Invest.*, **106** (suppl.), 167–171.

BRIZEL DM, KLITZMAN B, COOK JM, EDWARDS J, ROSNER G AND DEWHIRST MW. (1993). A comparison of tumor and normal tissue microvascular hematocrits and red cell fluxes in a rat window chamber model. *Int. J. Radiat. Oncol. Biol. Phys.*, **25**, 269–276.

BRIZEL DM, ROSNER GL, PROSNITZ LR AND DEWHIRST MW. (1995). Patterns and variability of tumor oxygenation in human soft tissue sarcomas, cervical carcinomas, and lymph node metastases. *Int. J. Radiat. Oncol. Biol. Phys.*, **25**, 1121–1125.

CHAPLIN DJ AND HILL SA. (1995). Temporal heterogeneity in microregional erythrocyte flux in experimental solid tumours. *Br. J. Cancer*, **71**, 1210–1213.

COLEMAN CN. (1988). Hypoxia in tumors: a paradigm for the approach to biochemical and physiologic heterogeneity. *J. Natl Cancer Inst.*, **80**, 310–317.

DEGNER FL AND SUTHERLAND RM. (1988). Mathematical modelling of oxygen supply and oxygenation in tumour tissues: prognostic, therapeutic, and experimental implications. *Int. J. Radiat. Oncol. Biol. Phys.*, **15**, 391–397.

DELIDES GS, VENIZELOS J AND RÉVÉSZ L. (1988). Vascularization and curability of stage III and IV nasopharyngeal tumors. *J. Cancer Res. Clin. Oncol.*, **114**, 321–323.

DEWHIRST MW, ONG ET, KLITZMAN B, SECOMB TW, VINUYA RZ, DODGE R, BRIZEL D AND GROSS JF. (1992). Perivascular oxygen tensions in a transplantable mammary tumor growing in a dorsal flap window chamber. *Radiat. Res.*, **130**, 171–182.

DEWHIRST MW, SECOMB TW, ONG ET, HSU R AND GROSS JF. (1994). Determination of local oxygen consumption rates in tumors. *Cancer Res.*, **54**, 3333–3336.

DISCHE S, ANDERSON PJ, SEALY R AND WATSON ER. (1983). Carcinoma of the cervix – anaemia, radiotherapy and hyperbaric oxygen. *Br. J. Radiol.*, **56**, 251–255.

GATENBY RA, KESSLER HB, ROSENBLUM JS, COIA LR, MOLDOFSKY PJ, HARTZ WH AND BRODER GJ. (1988). Oxygen distribution in squamous cell carcinoma metastases and its relationship to outcome of radiation therapy. *Int. J. Radiat. Oncol. Biol. Phys.*, **14**, 831–838.

GLASSBURN JR, BRADY LW AND PLENK HP. (1977). Hyperbaric oxygen in radiation therapy. *Cancer*, **39**, 751–765.

HENK JM, KUNKLER PB AND SMITH CW. (1977). Radiotherapy and hyperbaric oxygen in head and neck cancer. *Lancet*, **2**, 101–103.

HIRST DG. (1986). Oxygen delivery to tumours. *Int. J. Radiat. Oncol. Biol. Phys.*, **12**, 1271–1277.

HORSMAN MR. (1995). Nicotinamide and other benzamide analogs as agents for overcoming hypoxic cell radiation resistance in tumours. *Acta Oncol.*, **34**, 571–587.

HÖCKEL M, KNOOP C, SCHLENGER K, VORNDRAN B, BAUßMANN E, MITZE M, KNAPSTEIN PG AND VAUPEL P. (1993). Intratumoral $pO_2$ predicts survival in advanced cancer of the uterine cervix. *Radiat. Oncol.*, **26**, 45–50.

KOLSTAD P. (1968). Intercapillary distance, oxygen tension and local recurrence in cervix cancer. *Scand. J. Clin. Invest.*, **106** (suppl.), 145–157.

LAUK S, SKATES S, GOODMAN M AND SUIT HD. (1989). A morphometric study of the vascularity of oral squamous cell carcinomas and its relation to outcome of radiation therapy. *Eur. J. Cancer Clin. Oncol.*, **25**, 1431–1440.

LYNG H, MONGE OR, BØHLER PJ AND ROFSTAD EK. (1991). Temperature distribution in locally advanced breast carcinoma during hyperthermic treatment: relationship to perfusion, vascular density, and histology. *Int. J. Radiat. Oncol. Biol. Phys.*, **21**, 423–430.

LYNG H, SUNDFØR K, TROPÉ C AND ROFSTAD EK. (1995). Heterogeneity in $pO_2$ and histological appearance in human cervix carcinoma. In *Tumor Oxygenation*, Vaupel PW, Kelleher DK and Günderoth M (eds) pp. 249–258. Gustav Fischer Verlag: New York.

MOONEN CTW, VAN ZIJL PCM, FRANK JA, LE BIHAN D AND BECKER ED. (1990). Functional magnetic resonance imaging in medicine and physiology. *Science*, **250**, 53–61.

OKUNIEFF P, HOECKEL M, DUNPHY EP, SCHLENGER K, KNOOP C AND VAUPEL P. (1993). Oxygen tension distributions are sufficient to explain the local response of human breast tumors treated with radiation alone. *Int. J. Radiat. Oncol. Biol. Phys.*, **26**, 631–636.

OKUNIEFF P, DUNPHY E, HOECKEL M, DE BIE JJ, SCHLENGER K AND TERRIS DJ. (1995). Oxygenation and the response of patients to cancer therapy. *Abstract from the Ninth International Conference on Chemical Modifiers of Cancer Treatment*, 22–26 August 1995. pp. 263–264.

RÉVÉSZ L AND BALMUKHANOV SB. (1987). Anaemia as a prognostic factor for the therapeutic effect of radiosensitizers. *Int. J. Radiat. Biol.*, **51**, 591–595.

RÉVÉSZ L, SIRACKÁ E, SIRACKÝ J, DELIDES G AND PAVLAKI K. (1989). Variation of vascular density within and between tumors of the uterine cervix and its predictive value for radiotherapy. *Int. J. Radiat. Oncol. Biol. Phys.*, **16,** 1161–1163.

SECOMB TW, HSU R, ONG ET, GROSS JF AND DEWHIRST MW. (1995). Analysis of the effects of oxygen supply and demand on hypoxic fraction in tumors. *Acta Oncol.*, **34,** 313–316.

SIRACKÁ E, SIRACKÝ J, PAPPOVÁ N AND RÉVÉSZ L. (1982). Vascularization and radiocurability in cancer of the uterine cervix. *Neoplasma*, **29,** 183–188.

SIRACKÁ E, RÉVÉSZ L, KOVÁC R AND SIRACKÝ J. (1988). Vascular density in carcinoma of the uterine cevix and its predictive value for radiotherapy. *Int. J. Cancer*, **41,** 819–822.

STREFFER C, VAN BEUNINGEN D, GROSS E, EIGLER F-W AND PELZER T. (1989). Determination of DNA, micronuclei and vascular density in human rectum carcinomas. In *Prediction of Tumor Treatment Response*, Chapman JD, Peters LJ and Withers HR (eds) pp. 217–226. Pergamon Press: New York.

STONE HB, BROWN JM, PHILLIPS TL AND SUTHERLAND RM. (1993). Oxygen in human tumors: correlations between methods of measurement and response to therapy. *Radiat. Res.*, **136,** 422–434.

TANNOCK IF. (1972). Oxygen diffusion and the distribution of cellular radiosensitivity in tumours. *Br. J. Radiol.*, **45,** 515–524.

VAUPEL P. (1990). Oxygenation of human tumors. *Strahlenther. Onkol.*, **166,** 377–386.

VAUPEL P. (1993). Oxygenation of solid tumors. In *Drug Resistance in Oncology*, Teicher BA (ed.) pp. 53–85. Marcel Dekker: New York.

VAUPEL P, KALLINOWSKI F AND OKUNIEFF P. (1989). Blood flow, oxygen and nutrient supply, and metabolic microenvironment of human tumors: a review. *Cancer Res.*, **49,** 6449–6465.

WEIBEL ER. (1979). *Stereological Methods. Practical Methods for Biological Morphometry.* Vol 1. Academic Press: London.

### Appendix

The oxygen transport from a capillary into tumour tissue can be described in one dimension by:

$$P(x) = \frac{M}{2D\alpha}x^2 + \mathrm{A}x + \mathrm{B} \qquad (1)$$

where $P$ is tissue $pO_2$, $x$ is the distance from the capillary, $M$ is the oxygen consumption rate, $D$ is the diffusion coefficient and $\alpha$ is the solubility coefficient of oxygen in tissue, and A and B are constants depending on the boundary conditions (Dewhirst *et al.*, 1994). Equation (1) assumes that the oxygen diffusion occurs only in a plane through the capillaries and in the direction perpendicular to the vessels. Oxygen diffusion into tissue above and below the plane of the capillaries or diffusion resulting from gradients in $pO_2$ in the direction parallel to the vessels is not included in the equation. However, $pO_2$ values estimated from equation (1) correlate well with measured values (Dewhirst *et al.*, 1994).

Intracapillary $pO_2$ may be used as a measure of tumour $pO_2$ close to capillaries (Dewhirst *et al.*, 1992). The $pO_2$ profile can be calculated by assuming that the derivative of $P(x)$ is zero when $P(x)$ is zero (Tannock, 1972) and that the oxygen consumption rate is constant:

$$P(x) = \frac{M}{2D\alpha}x^2 - \sqrt{\frac{2MP_{cap}}{D\alpha}}x + P_{cap} \qquad (2)$$

where $P_{cap}$ is intracapillary $pO_2$.

A value of 2.0 $10^{-5}$ $cm^2$ $s^{-1}$ and 3.3 $10^{-5}$ $cm^3$ oxygen $cm^{-3}$ tissue $mmHg^{-1}$ was used for $D$ and $\alpha$ respectively (Degner and Sutherland, 1988). $P(x)$ halfway between two vessels was calculated for $x$ values of 95 $\mu m$ and 112 $\mu m$, corresponding to a vascular density of approximately 55 mm $mm^{-3}$ and 40 mm $mm^{-3}$ respectively.

**Acknowledgement**

Financial support from The Norwegian Cancer Society is gratefully acknowledged.